# Mechanism of Cs Immobilization within a Sodalite Framework: The Role of Alkaline Cations and the Si/Al Ratio

**DOI:** 10.3390/ijms242317023

**Published:** 2023-11-30

**Authors:** Anton Kasprzhitskii, Yakov Ermolov, Vasilii Mischinenko, Andrey Vasilchenko, Elena A. Yatsenko, Victoria A. Smoliy

**Affiliations:** 1 Technological Faculty, Platov South-Russian State Polytechnic University (NPI), Prosveshcheniya St., 132, Novocherkassk 346428, Russia; mr.yak.erm@gmail.com (Y.E.); m.vasbor@bk.ru (V.M.); zmandr@mail.ru (A.V.); e_yatsenko@mail.ru (E.A.Y.); vikk-toria@yandex.ru (V.A.S.); 2 Department of Civil Engineering, Rostov State Transport University, Narodnogo Opolcheniya Sq., Rostov-on-Don 344038, Russia; 3 Climate Center, Novosibirsk State University, Pirogov Street, 2, Novosibirsk 630090, Russia

**Keywords:** mesoporous materials, zeolite, geopolymer, immobilization, radioactive waste, Cs^+^, DFT

## Abstract

Conditioning of radioactive waste generated from the operation of medical institutions, nuclear cycle facilities, and nuclear facilities is important for the safety of the environment. One of the most hazardous radionuclides is radioactive cesium. There is a need for more effective solutions to contain radionuclides, especially cesium (Cs+). Geopolymers are promising inorganic materials that can provide a large active surface area with adjustable porosity and binding capacity. The existence of nanosized zeolite-like structures in aluminosilicate gels was shown earlier. These structures are candidates for immobilizing radioactive cesium (Cs^+^). However, the mechanisms of their interactions with the aluminosilicate framework related to radionuclide immobilization have not been well studied. In this work, the influence of alkaline cations (Na^+^ or K^+^) and the aluminosilicate framework structure on the binding capacity and mechanism of interaction of geopolymers with Cs^+^ is explored in the example of a sodalite framework. The local structure of the water molecules and alkaline ions in the equilibrium state and its behavior when the Si/Al ratio was changed were studied by DFT.

## 1. Introduction

Growing nuclear safety problems require more effective solutions for retaining radionuclides. Currently, a significant amount of low and intermediate-level radioactive waste has been accumulated (~24 million m^3^) [1,2]. One of the most hazardous radionuclides is radioactive cesium (^137^Cs). Its share in irradiated nuclear fuel is, on average, ~6 wt% [3]. Having a half-life of 30.7 years, it has strong radioactivity and high chemical reactivity, as well as good water solubility [4]. If released, Cs^+^ is capable of having a long-term detrimental effect on the environment. It can form aerosol particles, such as cesium hydroxide (CsOH), cesium carbonate (Cs_2_CO_3_), and cesium iodide (CsI). These particles contaminate soil and water by dry or wet deposition [5]. In this situation, it is essential to find a material to dispose of this radioactive waste safely. This material has to possess high radionuclide immobilization and sealing properties, providing a stable phase with low aqueous solubility and radionuclide leaching.

For a long time, cement-based materials, including ordinary Portland cement (OPC), cement–polymer composites, and other alternative binding systems were considered promising binders for sealing low- and intermediate-level radioactive wastes [6,7,8]. Cementation has significant advantages over other ways of conditioning radioactive waste, such as the thermal conversion of ceramics or glasses. It is characterized by low cost, availability of materials, and high productivity [9,10,11]. However, most cement-based materials are porous and relatively easily leached [12,13]. They are subject to structural degradation under acidic conditions and have low thermal stability [14]. This does not allow them to retain radionuclides in their structure for a long time [13]. The studies on immobilization of Cs^+^ ions in these materials showed a relatively high effective diffusivity (~10^−7^–10^−9^ cm^2^/s) [15,16]. In addition, Cs^+^ has good mobility, both in acidic and alkali conditions. This additionally increases its diffusivity [17,18].

Geopolymer binders, also known as alkali-activated binder materials or inorganic materials [19], are a promising alternative to OPC matrices [20]. They can be obtained by polycondensation in a strong alkaline solution [21]. Due to their ability to bind cations, high mechanical properties, and chemical durability, these materials have recently attracted considerable interest, particularly as materials suitable for the conditioning of radioactive waste [22,23,24]. This is indicated by a number of studies [25,26,27]. 

Thus, a significant decrease in ^137^Cs release was found during the physical immobilization of Cs-containing resin waste in a geopolymer matrix based on metakaolin [25]. The effect of increased immobilization was also observed for other radioactive resin wastes containing radionuclides ^134^Cs, ^60^Co, ^154^Eu, and ^152^Eu alkali-activated binders obtained from industrial by-products [26]. In addition, the use of crushed, granulated blast furnace slag to produce a geopolymer matrix makes it possible to increase the waste content of spent ion-exchange resin without reducing its immobilizing effect [27]. Such superiority in radionuclide immobilization by geopolymers is attributed to its reaction products. The existence of nanosized zeolite-like structures in aluminosilicate gels was shown earlier [28]. They can play an important role in immobilization by creating structural sites for the retention and accumulation of alkaline cations [29,30]. The structural framework of aluminosilicate gel produced using Na- and K-containing alkali is quasi-zeolite. In this structure, aluminum and silicon atoms are in tetrahedral coordination. A geopolymer is a network of interconnected silicate and aluminate groups. The structural coordination of aluminum and silicon depends on the Al/Si ratio with a restriction on Al-O-Al bond formation [30]. The unbalanced negative charge resulting from the presence of Al in the structure is balanced by alkali metal cations, providing the aluminosilicate gel with additional stability. Tetrahedral-bound aluminate groups are negatively charged and, therefore, can serve as cation-binding centers. These charge-compensating cations involved in the formation of the framework are alkali or alkaline-earth metal ions, such as Na^+^ and K^+^ (Na^+^ and K^+^). Cs^+^ can play the same role [30]. The properties of a geopolymer matrix vary depending on the composition of the initial aluminosilicate material [31,32,33], the nature of the alkali activation solution [34,35], and the curing conditions [36,37]. Its local structure is influenced by the ratios of silica and aluminum oxide in the precursors and also depends on the conditions of the alkaline activation reaction [38,39]. Besides, crystalline zeolite phases with long-range order are formed in the aluminosilicate gel structure at elevated temperatures [40]. The manifestation of these structural features is associated with the ratio of Si to Al. At high values of this ratio, a glass-like porous amorphous matrix is formed [32,41]. Conversely, low values (Si/Al < 2) lead to the formation of a composite structure with crystalline regions embedded in an amorphous matrix [30,42,43]. The crystalline regions are often represented by zeolites or similar structures [44,45]. These zeolite-like regions in the geopolymer structure provide excellent immobilizing properties and high cation exchange capacity [46,47]. 

The possibility of obtaining data on zeolite structures is important for the storage of radioactive waste. A number of studies have been performed using geopolymer materials for radioactive waste storage [48]. Metakaolin-based geopolymers have been shown to have high immobilization activity for heavy metal ions [49]. It has been found that there are several types of centers for heavy metal binding during adsorption [50]. Cs^+^ is the most abundant radionuclide studied. In addition, some other radionuclide cations can be incorporated into geopolymers [51,52]. Most studies on the ability of geopolymers to immobilize Cs^+^ have mainly focused on confirming the incorporation of the radionuclide into its structure, and the associated mechanism has been little studied [52,53,54]. The factors determining the long-term stability of radionuclide cations in the geopolymer structure also have not been sufficiently studied. In recent studies on the immobilization mechanism of heavy metal cations and radionuclides [55,56], the role of molar ratios (Si/Al) and the influence of the alkaline cation type (Na^+^/K^+^) on the Cs^+^ binding mechanism remained unexplored. 

This work used the DFT method to investigate the mechanism of Cs^+^ immobilization in the sodalite structure. Understanding this mechanism at the atomic level is a key step in a rational approach to optimizing the composition and structure of the geopolymer to maximize the confinement of the Cs^+^ cation.

## 2. Results and Discussion 

### 2.1. Local Structure of Cs^+^, Na^+^, and K^+^

**Figure 1:**
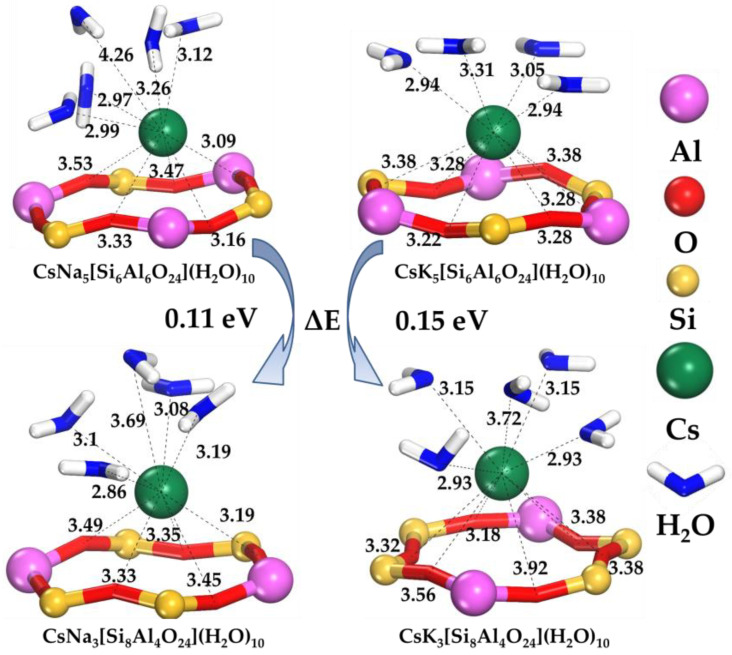
Structure of Cs^+^ immobilized in Na- and K-type sodalites. The distances between the Cs^+^ and oxygen atoms of the framework and water molecules are given in Å.

**Figure 2:**
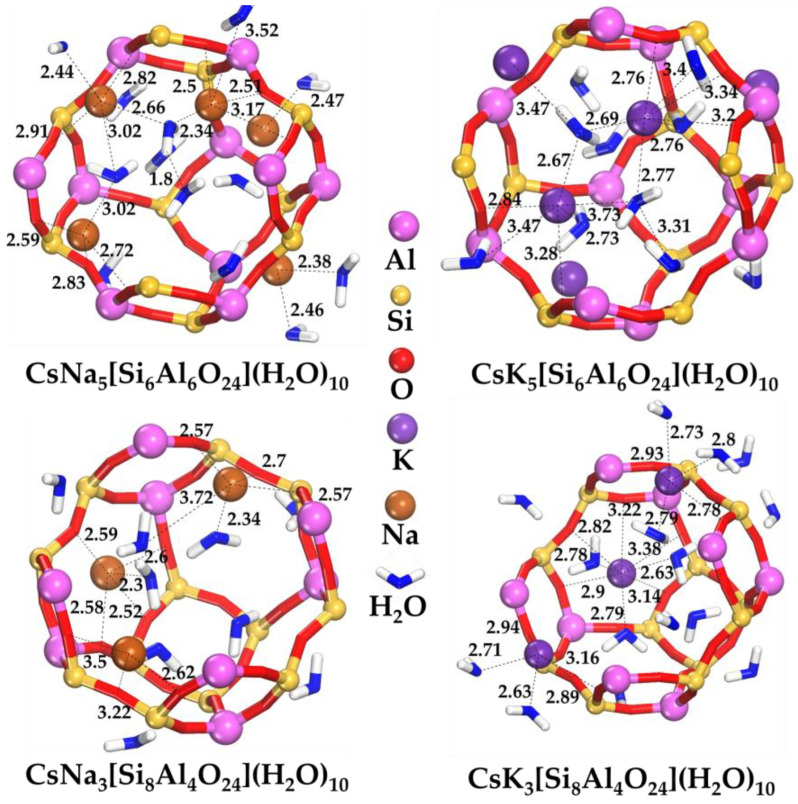
Distribution of Na^+^ and K^+^ in SOD structures. The distances between the Na^+^ or K^+^ and oxygen atoms of the framework and water molecules are given in Å.

The equilibrium configurations corresponding to the lowest energy after the sequential molecular dynamic and DFT calculations are shown in Figure 1 and Figure 2.

Figure 1 shows the solvated state of the radionuclide Cs^+^ after optimization in a sodalite framework. The position of the Cs^+^ ion is coordinated by a six-membered sodalite ring. Despite its flexibility, which is ensured by changing the Si-O and Al-O bonds, Cs^+^ is displaced from its center during the optimization process. In the equilibrium state, Cs^+^ is in the center of the region coordinated by the oxygen atoms of the SOD framework on one side and the oxygen atom of the water molecules on the other side. The average values of the distances between the above atoms are dependent on the factors studied. An increase in the Si/Al ratio leads to an increase in the average distances between Cs^+^ and the oxygen atoms of the SOD framework. Thus, the distance between the oxygen atoms of the SOD framework and Cs+ increases for Na-type sodalite from ~3.32 Å to ~3.47 Å. For K-type sodalite, the distances increase from ~3.28 Å to ~3.46 Å. These values of the Cs^+^-O distances agree with the data from [57,58].

The average distance between Cs^+^ and water molecules also rises from ~3.08 Å to ~3.18 Å for Na-type sodalite, whereas for K-type sodalite, it increases from ~3.06 Å to ~3.17 Å. In the case of K-type sodalite, a denser structure stabilizing the Cs^+^ position can be observed. In addition, the structure is more stable for both cases at the Si/Al = 1 structural ratio. This can be explained by the greater number of Na or K alkali ions stabilizing the framework structure compared to the Si/Al = 2 case.

The observed structural regularities determined on the basis of the analysis of the interatomic distances are in agreement with the energetic estimation of the calculated equilibrium states. The calculated energy change ∆*E* shows that for the K-type (${∆E}_{K}$) sodalite, the threshold between states with different Si/Al ratios is greater than for Na-type sodalite (${∆E}_{Na}$). In this case, the Cs^+^ radionuclide has a more stable state in the structure with Si/Al = 1. An increase in the Si/Al ratio leads to an increase in the energy of the system, moving the Cs^+^ radionuclide to a less stable state. Taking into account the difference in the value of ∆E for K-type (~0.15 eV) and Na-type sodalites (~0.11 eV), the K-type sodalite system with Si/Al = 1 has a more stable Cs^+^ state. This is consistent with the findings of [59].

Figure 2 shows the equilibrium states of the Na^+^ and K^+^ alkaline ions in the sodalite framework. The Na^+^ and K^+^ ions are predominantly centered by the six-membered sodalite ring. They are also solvated by three water molecules. As the Si/Al ratio increases, the average distances between the alkaline ion and oxygen atoms of the sodalite framework increase by ~0.09 Å for the Na-type sodalite and by ~0.03 Å for the K-type sodalite. There is also a regular increase in the distance between the water molecules and the Na^+^ ion by ~0.04 Å and the K^+^ ion by ~0.03 Å. These regularities are fully determined by the Si/Al ratio.

### 2.2. Charge Transfer

**Figure 3:**
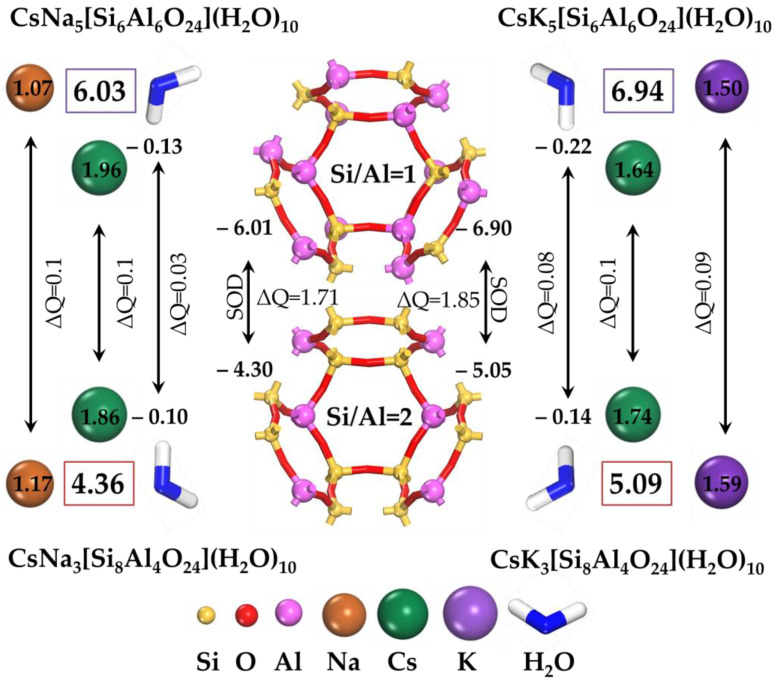
Mulliken charges and their changes are given for alkaline Cs^+^, Na^+^, and K^+^ ions as well as H_2_O.

The peculiarities of the behavior of the radionuclide Cs^+^ and the stability of its position were examined on the basis of the analysis of Mulliken atomic charges (Q) and the value of its change (ΔQ) [60]. A positive value of charge change (ΔQ) means a loss of electrons, and a negative value means their gain. The results of the state estimation of the lowest energy configurations are shown in Figure 3.

In addition to the aluminosilicate framework, the molecules of water and alkaline ions play an important role in the stability of the Cs^+^ position. The possibility structural rearrangement of water molecules will allow them to assume positions that provide increased stability of the aluminosilicate framework, supported by research data [61,62]. This is explained by the fact that water molecules receive a negative charge in the modeled system, acting as an additional framework. By increasing the distribution of negative charge in a given volume, water molecules increase the stability of the whole aluminosilicate structure. In this case, positively charged alkaline cations Na^+^, K^+^, and Cs^+^ compensate for the negative charge, not only of the SOD framework but also of the water molecules (Figure 3).

Thus, the average charge received by the water molecule in the K-type sodalite is greater than that of the Na-type sodalite. At a Si/Al = 1 ratio for K-type sodalite, water molecules have a negative charge of ~2.2 e and for Na-type, ~1.3 e. At the same time, for Si/Al = 2, it reaches ~1.4 e (K-type sodalite) and ~1 e (Na-type sodalite) per water molecule. That is, the charge decreases as the Si/Al ratio increases. The combined negative charge created by the aluminosilicate SOD framework and water molecules reduces the mobility of alkaline ions, increasing the immobilizing effect.

In the case of Si/Al = 1, the charge value of the molecules of water and alkaline ions in the K-type sodalite system (~6.94 e) is greater than that in the Na-type sodalite (~6.03 e). It decreases proportionally with a change in the Si/Al = 2 ratio to ~5.09 e (K-type sodalite) and ~4.36 e (Na-type sodalite). This indicates that K-type sodalite is a more stable system than Na-type sodalite due to its higher absolute charge values. Meanwhile, the stability of the system decreases with increasing Si/Al according to the following sequence: K-type sodalite > Na-type sodalite. In this case, K-type sodalite has a greater ability to retain the radionuclide Cs^+^ than a Na-type sodalite system.

### 2.3. Density of Electronic States

**Figure 4:**
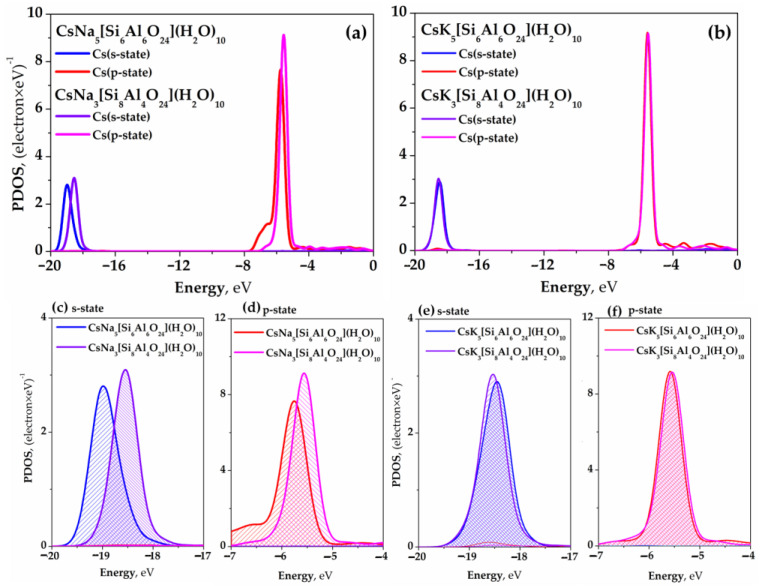
PDOS of Cs^+^ for the Na- and K-type sodalities: (**a**) PDOS of Cs^+^ for the Na-type sodalite; (**b**) PDOS of Cs^+^ for the K-type sodalite; (**c**) PDOS (s-state) of Cs^+^ for the Na-type sodalite; (**d**) PDOS (p-state) of Cs^+^ for the Na-type sodalite; (**e**) PDOS (s-state) of Cs^+^ for the K-type sodalite; (**f**) PDOS (p-state) of Cs^+^ for the K-type sodalite.

The influence of the type of SOD framework with a given ratio (Si/Al) and alkaline cation (Na^+^ or K^+^) on the stability of the Cs^+^ state was studied based on its PDOS analysis. The partial density of electron states (PDOS) determines the number of electron states per unit of energy. The PDOS region of the radionuclide Cs^+^ for s-symmetry (from −20 to −17 eV) and p-symmetry (from −7 to −4 eV) for the Na-type and K-type sodalites is shown in Figure 4a–f.

Figure 4a presents the PDOS of Cs^+^ for the Na-type sodalite case when the Si/Al ratio is changed. For the Si/Al = 1 case, the electronic states of Cs^+^ are low-energy. They are in a more energetically favorable position compared to the Si/Al = 2 case. The difference between the states is ~0.4 eV for s-symmetry (Figure 4c), whereas for p-symmetry it is ~0.2 eV (Figure 4d). For the K-type sodalite (Figure 4b), the energetic position of the PDOS of Cs^+^ for different Si/Al values remains practically unchanged for the s-symmetry (Figure 4e) and p-symmetry (Figure 4f) states. The presence of the K^+^ alkaline ion in the SOD structure has a stabilizing effect on the electronic states of Cs^+^ when the Si/Al ratio changes, in contrast to the Na-type sodalite. This result aligns with data on the charge change in water molecules and alkaline ions observed for these cases (see Section 2.2).

### 2.4. Electron Density Difference

The nature of the interactions among the SOD framework, water molecules, alkaline cations (Na^+^, K^+^), and Cs^+^ can be established on the basis of an analysis of electron transfer between neighboring atoms in the modeled system. The difference in electron density allows for estimating the redistribution of electrons and determining the interactions between atoms in the aluminosilicate framework.

**Figure 5:**
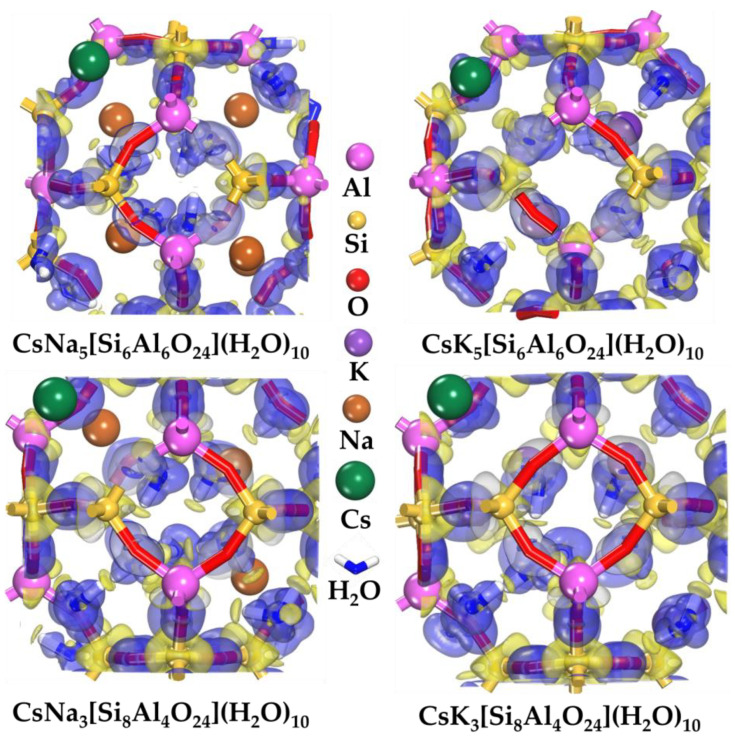
The electron density differences of Cs^+^ and H_2_O for the Na- and K-type sodalites. The isosurface value is 0.1 electrons/Å^3^, where the blue and yellow areas denote the electron accumulation and the electron depletion, respectively.

The electron density differences for the structure of Cs^+^ immobilized in Na- and K-type sodalites are presented in Figure 5.

In Figure 5, the yellow-colored areas indicate the loss of charge, and the blue-colored areas indicate its accumulation. The observed pattern of electron density changes is consistent with the charge transfer data (see Section 2.2). The centers of charge accumulation in the SOD framework are oxygen atoms, which receive charge to a greater extent from Si atoms and to a lesser extent from Al atoms. At the same time, the oxygen atoms of the SOD framework bond to the hydrogen of water molecules, forming hydrogen bonds. Hydrogen bonding is partially covalent in nature due to the electron donor–acceptor interaction between the electron donor—hydrogen atom of water molecules and the electron acceptor—oxygen atom of the SOD framework. The increase of electron density on the oxygen atom occurs through an intermediary—hydrogen bridge. In this case, partial filling of the non-bonding orbital of the hydrogen atom is allowed. A characteristic charge transfer is observed, accompanied by a depletion of electron density at the H atom and its accumulation by the O atom. At the same time, hydrogen bonds are formed both between the SOD framework and water molecules and between the molecules themselves. This network of hydrogen bonds provokes structural rearrangements of water molecules, providing additional stability of the resulting system. The resulting pattern of electron density changes is consistent with the transferred charge between the SOD framework and water molecules (see Section 2.2). Thus, water molecules act as an additional rearrangeable framework, reinforcing the SOD framework through hydrogen bonding.

## 3. Materials and Methods

A sodalite (SOD) framework was chosen as a model to study the mechanism of Cs^+^ immobilization [63]. SOD is a characteristic structural element for zeolites [64], occurring in an amorphous matrix in geopolymers derived from metakaolin [65]. Generally, the composition of zeolites is close to that of the aluminosilicate gel from which they crystallize. The occurrence of zeolite A is observed in geopolymers predominantly at a Si/Al ≈ 1 ratio [66]. It was demonstrated that it is possible to form zeolites of the phojasite structure at Si/Al ratios above one [67]. It was also shown that zeolites can be formed at higher Si/Al ratios: Na-chabazite (Si/Al ≈ 2.6) [68], hydroxysodalite (Si/Al ≈ 2.46), zeolite A (Si/Al ≈ 3) [68], and faujasite (Si/Al ≈ 2) [69]. The initial SOD silicate framework was modified by replacing Si atoms with Al atoms following the Loewenstein rule with a given Si/Al ratio (~1 or 2) [70]. 

**Figure 6:**
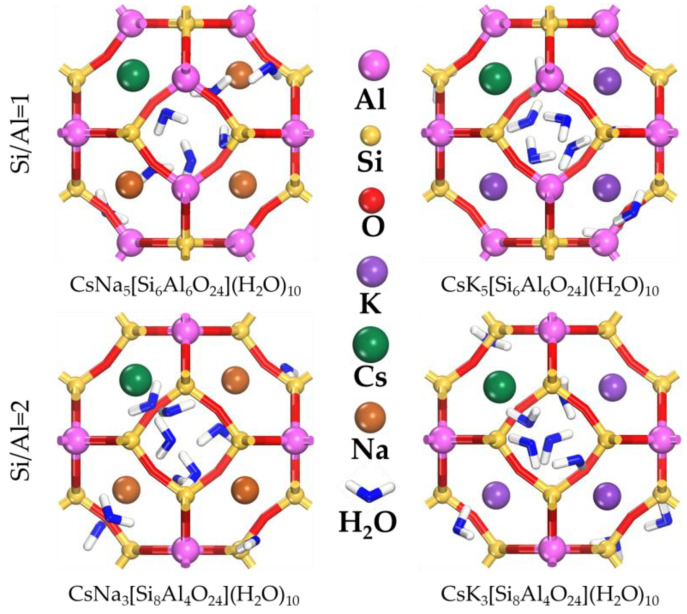
Model of sodalite with different molar ratios of Si/Al.

The number of added alkali metal ions (Na^+^, K^+^, and Cs^+^) equals the number of Al atoms to maintain charge neutrality according to the selected cases (Figure 6). The selected number of water molecules (10H_2_O) corresponds to earlier estimates [71]. The loading of the water and ions into the unit cell was performed using the RASPA [72]. The loading process included 4,000,000 steps for equilibrating the water and ions into the framework, followed by 10,000,000 steps for the ensemble average. The ClayFF force field was used to calculate the full energies of the equilibrium states. ClayFF uses a set of parameters that include partial atomic charges, van der Waals forces, and angular and covalent bonding parameters [73]. These parameters have been optimized to achieve realistic simulations of the structure and dynamics of clay minerals and their interactions with water molecules. ClayFF has been widely applied in the study of aluminosilicate minerals in geology, materials science, and chemistry. It is particularly useful in studies related to adsorption and diffusion in aluminosilicate materials as well as in studies of their properties [74]. In order to obtain the most stable and in equilibrium structure after the water and ions loading step in the SOD framework model, molecular dynamic simulation was performed. 

The structure was equilibrated at P = 1 atm and T = 298 K for 1 ns in an NPT ensemble with periodic boundary conditions imposed on the cell. The ClayFF force field was used for the simulation procedure [73]. The Verlet time integration scheme and a time step of integration of 0.1 fs were used. The Verlet time integration scheme, widely used in molecular dynamics simulations, offers several key advantages that make it a popular choice in computational physics and chemistry. The algorithm is computationally efficient, requiring a minimal number of force evaluations per time step compared to other integration methods. It conserves the total energy of a system very well over a long time, which is crucial for the physical accuracy of simulations of molecular dynamics [75].

The simulation was performed using the Nose thermostat and Parrinello-Rahman barostat. The Ewald and atom summation method was used for electrostatic and van der Waals interactions. Subsequent optimization of the DFT geometry was performed for each of the obtained models of the aluminosilicate framework with molecules of water and alkaline ions loaded into the cell. The atomic coordinates of all atoms in the cell were optimized. 

All DFT calculations were performed using the Cambridge Serial Total Energy Package (CASTEP) code, which uses a combination of plane waves and pseudopotentials [76] and the imposition of periodic boundary conditions [77] on the simulated cell. CASTEP allows accurate first-principles quantum mechanical calculations of the properties of molecules, crystals, and surfaces. It is widely used in materials science, chemistry, and solid-state physics to investigate the properties of new materials. The exchange and correlation interactions were described in the generalized-gradient approximation [78] using the Perdew–Burke–Ernzerhof (PBE) functional [79]. The PBE provides a good balance between accuracy and computational efficiency. The choice of this functional is based on the fact that it accurately describes the interactions of an aluminosilicate framework with molecular systems [80]. The calculations were performed using the ultrasoft pseudopotential [81], which provides high accuracy and counting speed [82] and has a wide range of applications [83,84]. The plane–wave energy cutoff was determined to be 1000 eV, and electron states in the Brillouin zone were sampled using a 4 × 4 × 4 k-point grid using the Monkhorst–Pack scheme. The Monkhorst–Pack scheme ensures an optimal balance between computational load and accuracy of results. This scheme facilitates precise calculations of band structures, allowing researchers to thoroughly analyze the electronic properties of materials [85]. 

The calculations took into account the following valence states of the atoms of the simulated systems: Si(3s^2^3p^2^), Al(3s^2^3p^1^), O(2s^2^2p^4^); H(1s^1^), Na(2s^2^2p^6^3s^1^), K(3s^2^3p^6^4s^1^), and Cs(5s^2^5p^6^6s^1^). Optimization of the cell parameters and positions of the atoms in it was performed using the Broyden, Fletcher, Goldfarb, and Shannon (BFGS) algorithm. The BFGS belongs to the class of quasi-Newton methods for solving unconditional optimization problems. This algorithm uses information about the gradient (first derivative of the function) and the approximate value of the inverse Hesse matrix to update the estimates of the optimization variables at each step. It usually provides good convergence to a local extremum, especially if the initial approximation is close enough to the optimized solution [86]. The following convergence criteria were established to determine the energetic position of the atoms in the cell: 5 × 10^−4^ Å for maximum displacement, 0.02 GPa for maximum stress, 0.01 eV/Å for maximum force, and 5 × 10^−6^ eV/atom for total energy. The self-consistency procedure to find the minimum energy at each step of the atomic position variation was performed until the accuracy of 5 × 10^−7^ eV/atom had been achieved.

To estimate the energy stability of the Cs^+^ radionuclide state and to determine the influence of the structure with a given Si/Al ratio, the calculation was performed according to the following formula:

(1) $$E_{Na/K}^{1}=E(Cs{\left[Na/K\right]}_{5})-E({\left[Na/K\right]}_{6}),$$

(2) $$E_{Na/K}^{2}=E(Cs{\left[Na/K\right]}_{3})-E({\left[Na/K\right]}_{4}),$$

(3) $${∆E}_{Na/K}=E_{Na/K}^{1}-E_{Na/K}^{2},$$

where $E(Cs{\left[Na/K\right]}_{5(3)})-$ total energy for the case Si/Al = 1(2).

The change in the charge of an atom is determined by the formula:

(4) $$∆q_{i}=q_{i,2}-q_{i,1},$$

where $q_{i,1}\;or\;q_{i,2}$—atomic charge for the case Si/Al = 1(2), respectively.

The electron density difference was determined as follows:

(5) $$∆\rho =\rho_{H_{2}O/SOD}-\rho_{H_{2}O}-\rho_{SOD},$$

(6) $$∆\rho =\rho_{Cs[Na/K]/SOD}-\rho_{Cs[Na/K]}-\rho_{SOD},$$

where $\rho_{H_{2}O/SOD}$, $\rho_{Cs[Na/K]/SOD}$, $\rho_{H_{2}O}$, $\rho_{Cs[Na/K]}$ and $\rho_{SOD}$ are the electron densities of the Cs[Na/K]/SOD system after immobilization, the free water molecule, and the SOD framework before immobilization, respectively.

## 4. Conclusions

In the present work, a DFT study of the mechanism of Cs^+^ binding to a sodalite framework was carried out. The influence of the alkaline cation type (Na^+^ or K^+^) and the aluminosilicate framework on Cs^+^ immobilization was studied. K-type sodalite was found to have a greater stabilizing effect on Cs^+^ than Na-type sodalite. The centers of charge accumulation in the SOD framework are oxygen atoms, which receive charge to a greater extent from Si atoms and to a lesser extent from Al atoms. The oxygen atoms of the SOD framework bond to the hydrogen of water molecules, forming hydrogen bonds. A characteristic charge transfer is observed, accompanied by the depletion of electron density at the H atom and its accumulation by the O atom. Hydrogen bonds are formed both between the SOD framework and water molecules and between the water molecules themselves. The possibility of structural rearrangement of water molecules allows them to occupy positions that provide increased stability of the aluminosilicate framework. An increase in the Si/Al ratio decreases the immobilizing effect for Cs^+^, which is caused by a reduction in the number of compensating alkaline ions, reducing the absolute values of the arising charges. The results of this study are consistent with the available experimental data.

## Data Availability

The data presented in this study are available upon request from the corresponding author.
